# Quantifying cellular dynamics in mice using a novel fluorescent division reporter system

**DOI:** 10.3389/fimmu.2023.1157705

**Published:** 2023-07-27

**Authors:** Eva Lukas, Thea Hogan, Cayman Williams, Benedict Seddon, Andrew J. Yates

**Affiliations:** ^1^ Department of Pathology and Cell Biology, Columbia University Irving Medical Center, New York, NY, United States; ^2^ Theoretical Biology and Bioinformatics, Department of Biology, Utrecht University, Utrecht, Netherlands; ^3^ Institute of Immunity and Transplantation, Division of Infection and Immunity, University College London (UCL), Royal Free Hospital, London, United Kingdom

**Keywords:** naive T cells, mathematical model, immunology, mouse model, dynamics

## Abstract

The dynamics of cell populations are frequently studied *in vivo* using pulse-chase DNA labeling techniques. When combined with mathematical models, the kinetic of label uptake and loss within a population of interest then allows one to estimate rates of cell production and turnover through death or onward differentiation. Here we explore an alternative method of quantifying cellular dynamics, using a cell fate-mapping mouse model in which dividing cells can be induced to constitutively express a fluorescent protein, using a Ki67 reporter construct. We use a pulse-chase approach with this reporter mouse system to measure the lifespans and division rates of naive CD4 and CD8 T cells using a variety of modeling approaches, and show that they are all consistent with estimates derived from other published methods. However we propose that to obtain unbiased parameter estimates and full measures of their uncertainty one should simultaneously model the timecourses of the frequencies of labeled cells within both the population of interest and its precursor. We conclude that Ki67 reporter mice provide a promising system for modeling cellular dynamics.

## Introduction

Describing the dynamics of cell populations – their rates of production from precursors and self-renewal, and their rate of loss – is important for understanding how homeostasis is maintained and how these populations respond to perturbations. Many studies of cellular dynamics employ DNA labeling, in which an identifiable agent such as bromodeoxyuridine (BrdU), or deuterium derived from deuterated glucose or heavy water, is taken up by dividing cells. Tracking the increase in frequency of labeled cells during a period of label administration, and its decline afterwards, allows one to measure rates of production and turnover, using mathematical models (1, 2).

Recently, there has been growing use of Cre recombinase activated fluorescent fate-reporters in mice to study immune cell homeostasis. In these systems, tamoxifen inducible CreERT expression is controlled in a manner dependent upon a specific developmental stage or lineage specification. When expressed alongside Cre reporter constructs, such as *Rosa26* ^RYFP^alleles, Cre recombinase excises transcriptional stop sequences in reporter constructs, resulting in the permanent and heritable expression of fluorescent reporters. Foxp3-CreERT mice have been used to label and track Foxp3 expressing cells and demonstrate that the Foxp3^+^ regulatory T cell phenotype and state is stable over time (3). In another study, elegant use of a Cd4-CreERT reporter strain permitted labeling and tracking of cohorts of CD8 lineage T cells, induced to express a Cre construct during their development as CD4^+^CD8^+^ thymic precursors (4). In general, these fate-reporter systems allow us to measure the rates at which cells make decisions during the period of induction of Cre recombinase, and to track their fates. If the gene of interest is specific to cell division, one could then in principle use a reporter system of this kind to quantify cell population dynamics.

**Figure 1:**
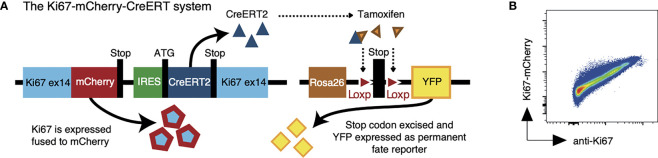
The Ki67 reporter system. **(A)** Schematic of the mouse model that allows us to follow recent cell division events with a fluorescent Ki67 reporter (mCherry), and also to indelibly label dividing cells and their progeny with yellow fluorescent protein (YFP). **(B)** Density plot shows Ki67-mCherry reporter expression vs anti-nuclear Ki67 mAb staining on total thymocytes.

We have reported the generation of a fate reporter strain, *Ki67-mCherry-CreERT* mice (5), in which the endogenous *Mki67* gene locus is modified to give rise to a Ki67-mCherry fusion protein, and a CreERT2 construct (**Figure 1A**). Ki67 is a nuclear protein which is expressed at peak levels during the cell cycle and is detectable for 3-4 days after mitosis (6–8). In these mice, active cell division can be monitored by mCherry fluorescence, which permits live cell sorting based on Ki67 expression (**Figure 1B**). In addition, in combination with a *Rosa26^RYFP^Cre* reporter construct, *Cre*activity in *Ki67-mCherry-CreER Rosa26* ^RYFP^ mice can be induced by feeding mice tamoxifen, to indelibly mark dividing cells and their progeny with YFP. Expression of mCherry and YFP therefore reports both a cell’s current and past division activity. Tamoxifen has a lifetime of a few hours *in vivo* (9), so varying the onset and duration of the tamoxifen pulse has the potential to track the fates of cells dividing within well-defined time windows, in an analogous way to pulse-chase DNA labeling assays. Previously we showed that the indelible and heritable expression of the Ki67 reporter allows one to track the fate of divided cells and their offspring over long timescales (5), and that the frequency of reporter expression in different populations provided an indication of the extent of their division activity. However this was not formally calibrated.

This study had two aims. First, to validate the use of this division reporter system to quantify rates of cell division and loss. Second, to explore how to maximize the information gained from YFP and Ki67 labeling timecourses, by implementing different strategies for dealing with potential sources of noise and bias in the data. To address both issues we used these reporter mice to study the dynamics of naive CD4 and CD8 T cells at steady state, because we and others have characterized the behavior of these populations extensively (7, 10–14). Briefly, the maintenance of large populations of naive T cells provides the basis of protection against novel pathogens (15). The diversity of the naive T cell repertoire is supplemented by continued production of new T cells from the thymus (11), and any proliferative renewal of existing naive T cells acts to preserve it (16). Quantifying such cellular dynamics is therefore important for understanding how naive T cell numbers and repertoire diversity are established and maintained (17–20), and more generally for understanding how the immune system reconstitutes following hematopoietic stem cell transplantation (21) or treatment of HIV infection (22).

## Results

### Ki67-mCherry-CreERT drives YFP reporter expression with low but predictable efficiency

**Figure 2:**
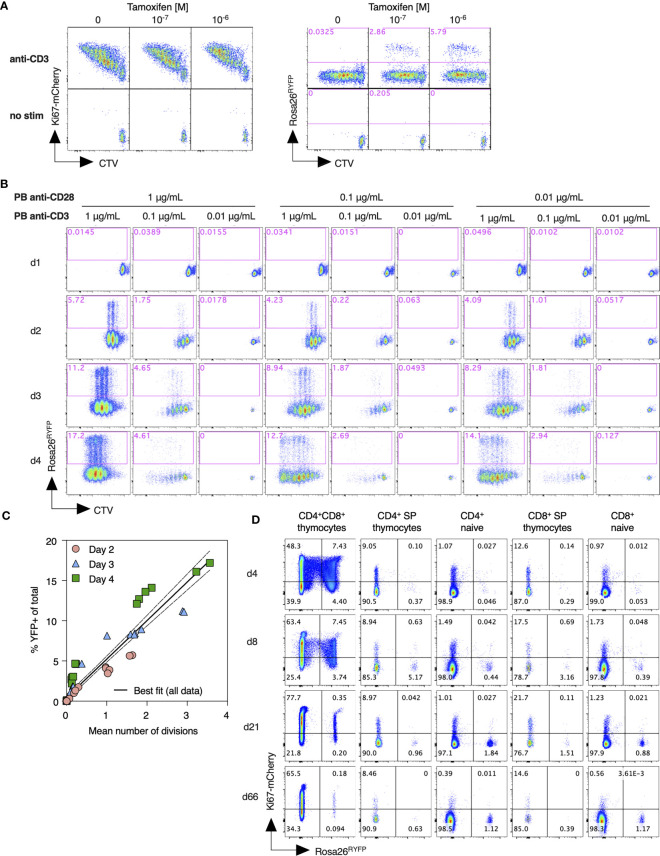
Assessment of Ki67 reporter activity and its relation to cell division. **(A)** Lymph node cells from *Ki67-mCherry-CreERT Rosa26^RYFP^* mice were labeled with CTV cell dye, and stimulated *in vitro* with plate bound anti-CD3 for 72h, with the indicated concentrations of tamoxifen (TAM). Density plots show Ki67-mCherry or YFP reporter expression vs CTV dilution by total TCR^+^ cells, as compared with unstimulated control cultures. **(B)** Lymph node cells from *Ki67-mCherry-CreERT Rosa26^RYFP^* mice were labeled with CTV, stimulated with the indicated concentrations of plate bound anti-CD3 and anti-CD28, and expression of YFP reporter vs CTV dilution analyzed at the indicated days. **(C)** %YFP-expressing cells from individual cultures vs mean division number, normalized for expansion, as determined by the CTV dilution profiles. Solid and dashed lines indicate the best fit linear relationships and 95% confidence envelope. **(D)** *Ki67-mCherry-CreERT Rosa26^RYFP^* mice were injected daily with tamoxifen for five consecutive days. Thymus and lymph nodes were recovered at the indicated times after starting injections, and expression of Ki67-mCherry and Rosa26 RYFP reporter measured amongst DP, CD4 SP, and CD8 SP thymocytes, and naive CD4 and CD8 T cells.

In order to better characterize the regulation of *Rosa26^RYFP^* reporter expression by Ki67 driven *Cre* activity, we first analyzed the tamoxifen-driven induction of the YFP reporter in dividing T cells *in vitro*. T cells from *Ki67-mCherry-CreER Rosa26^RYFP^* mice were labeled with CTV cell dye, to allow tracking of cell divisions, and activated *in vitro* by cross linking CD3 and CD28 receptors with plate bound mAb. Dividing T cells expressed high levels of Ki67-mCherry. A small subpopulation of dividing cells also induced YFP expression, that was strictly dependent upon 4OH-tamoxifen application and the dose thereof (**Figure 2A**). To assess the relation of YFP induction to cell division activity, we stimulated T cells under a range of conditions and measured CTV dye dilution profiles and YFP reporter induction over the following 4 days. These different culture conditions resulted in diverse proliferative activity and induction of YFP expression (**Figure 2B**). A direct comparison of YFP induction frequency with mean cell divisions within different cultures revealed a strong correlation that was largely independent of conditions of stimulation or the time at which cultures were analyzed (**Figure 2C**). Taken together, these data show that YFP reporter induction is strictly dependent upon cell division, and that the frequency of YFP induction in a given population is predictive of the average cell division activity.

Next, we analyzed induction of *Rosa26^RYFP^* reporter *in vivo*, following treatment of *Ki67-mCherry-CreER Rosa26 ^RYFP^* mice with intraperitoneal injection of 3mg tamoxifen for five consecutive days. Ki67 expression amongst naive T cells was very low, while thymocytes, which divide extensively during development, expressed it at much higher frequencies (**Figure 2D**). Therefore, we reasoned that inducing Cre activity by administration of tamoxifen should preferentially activate *Rosa26^RYFP^ Cre* reporter expression in thymocytes relative to peripheral naive T cells. Shortly after the fifth injection (d4), approximately 12% of CD4^+^CD8^+^ double positive (DP) thymocytes had induced expression of YFP (**Figure 2D**), while the frequencies of post-selection single positive thymocytes and peripheral naive T cells expressing YFP were very low. In the weeks following tamoxifen administration the fraction of cells expressing the YFP reporter dwindled amongst DP thymocytes, and gradually accumulated within peripheral naive subsets, consistent with the prediction that thymocytes time stamped by YFP induction would continue development and emerge into the peripheral naive pools.

### Estimates of mean residence times of naive T cells using reporter mice align with previous studies

To explore the use of the *Ki67-mCherry-CreERT* reporter mouse to study cell population dynamics at steady-state, we measured the numbers of YFP^+^ T cells in 61 mice aged between 42 and 110 days that underwent the 5 days of tamoxifen treatment (**Figure S1**). Mice were sacrificed at a range of timepoints across the following 62 days, and T cell subsets were harvested from thymi, lymph nodes and spleens. We then measured numbers of mature single positive CD4 (mSP4) and CD8 (mSP8) thymocytes, naive (CD44^low^CD62L^high^) CD4 and CD8 T cells within spleen and lymph nodes (**Figures S2A–C**), and the frequencies of YFP and Ki67-mCherry expression within these populations (**Figures S2D–I**). Naive T cells in mice recirculate freely between spleen and lymph nodes via the blood on timescales of hours (23–25), and Ki67 expression levels within each are strongly correlated (**Figure S2J**). We therefore proceeded by pooling the cell numbers from spleen and lymph nodes, and reporting the Ki67 and YFP expression fractions within the pooled population as the weighted averages of the measurements in spleen and lymph nodes. The sizes and Ki67 expression levels of these subsets in the treated reporter mice were indistinguishable from those of untreated reporter mice and of age-matched wild-type controls (**Figure S3**), indicating that both the YFP reporter constructs themselves and short-term tamoxifen treatment had no detectable effect on naive T cell dynamics in bulk.

**Figure 3:**
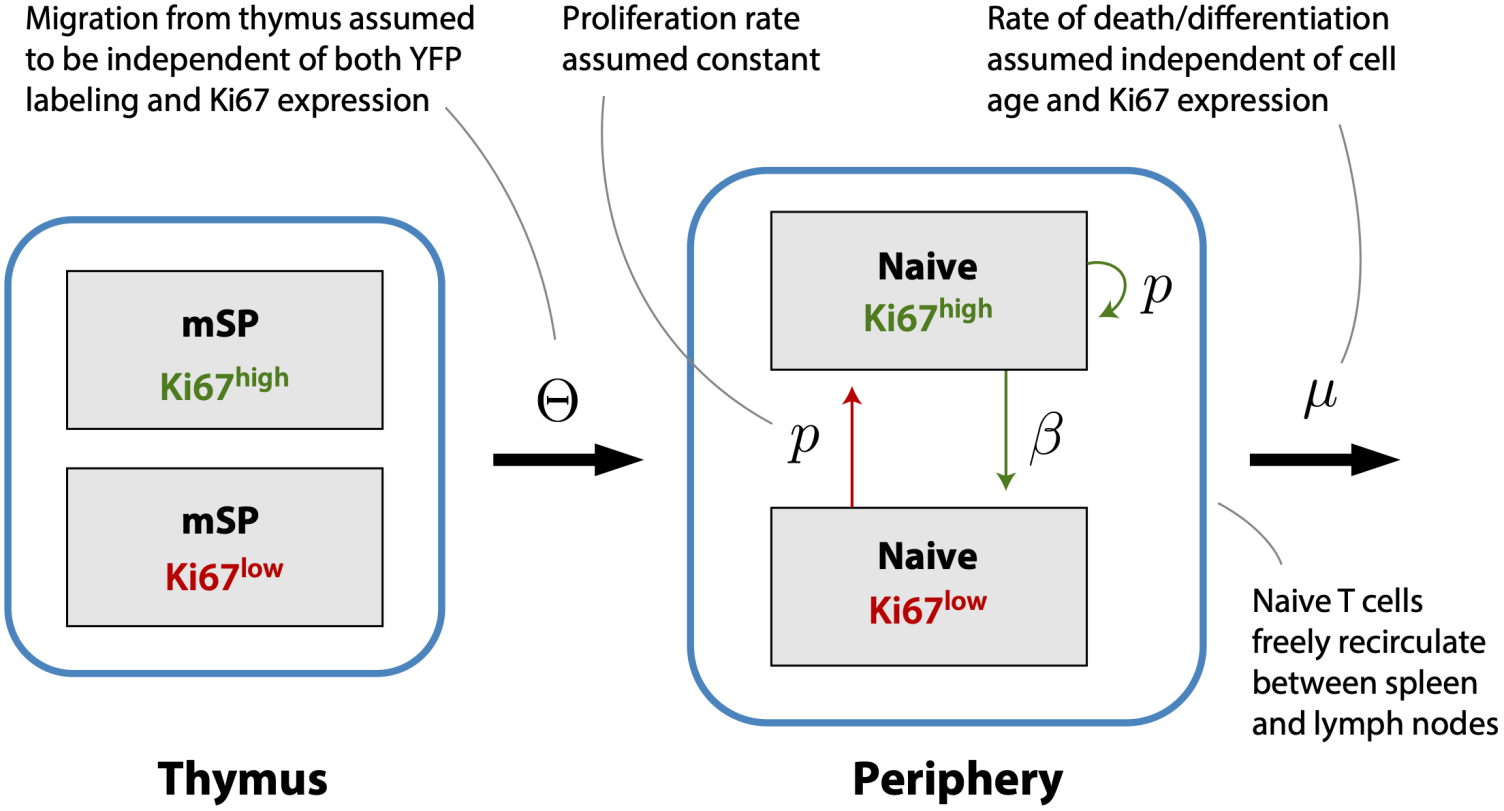
A simple homeogeneous model of naive T cell dynamics. New Ki67^+^ and Ki67^-^ naive T cells are exported from the thymus at rates proportional to the numbers of mature single positive Ki67^+^ and Ki67^-^ thymocytes, respectively. Once exported, naive T cells circulate between spleen and lymph nodes, are lost at rate *μ*, and divide at rate p. Division is accompanied by induction of Ki67 expression which persists for a mean time 1/*β* before cells become Ki67^-^.

Previous studies have shown that the expected residence times of naive CD4 and CD8 T cells increase slowly with their post-thymic age (4, 13, 14), and that thymic output in adult mice declines slowly, halving roughly every 6 months from approximately 8 weeks of age (7). (We refer to a cell’s ‘residence time’ within circulation, rather than ‘lifespan’, because the rate of loss of naive T cells combines death and onward differentiation.) In this experiment mice were sacrificed non-sequentially with respect to their age at the beginning of treatment, confounding the analysis of any age effects. However, the 70-day age range of mice is small compared to the timescales of change of thymic influx and of population average residence times, and so we did not expect to see a significant systematic impact of host and/or cell age on these dynamics. Instead, we assumed that naive CD4 and CD8 T cells could be considered broadly homogeneous with respect to their total rates of loss through death or onward differentiation, *μ*. We then constructed a model describing the kinetics of labeled cells within the thymic and peripheral naive T cell populations, stratifying each by Ki67 expression (**Figure 3**). This model assumes that self-renewal involves a naive T cell undergoing a single division with a low daily probability, then returning to a quiescent state, rather than a burst of divisions. Evidence for this mode of behavior comes from modeling of dye dilution assays of naive T cell proliferation under lymphopenic conditions (17).

Several studies have inferred that naive T cells in mice self-renew rarely, if at all (7, 10–14). We therefore began by exploring a reduced model in which the division rate *p* was set to zero and we ignored Ki67 expression. In this model, any YFP expression within the naive population derives from cells that divided in the thymus during tamoxifen treatment and were subsequently exported. We assume that mSP cells leave the thymus at a constant *per capita* rate Θ, motivated by the observation that the total rate of thymic output scales linearly with thymocyte numbers (26). Labeled naive T cells *(L)* in spleen and lymph nodes are lost at per capita rate *μ*. This rate is assumed to be identical for labeled and unlabeled cells. If the number of YFP^+^ mSP4 or mSP8 cells is *Y (t)*, then;


(1) $$\frac{dL}{dt}=\text{Θ}Y\left(t\right)-\mu L.$$

**Figure 4:**
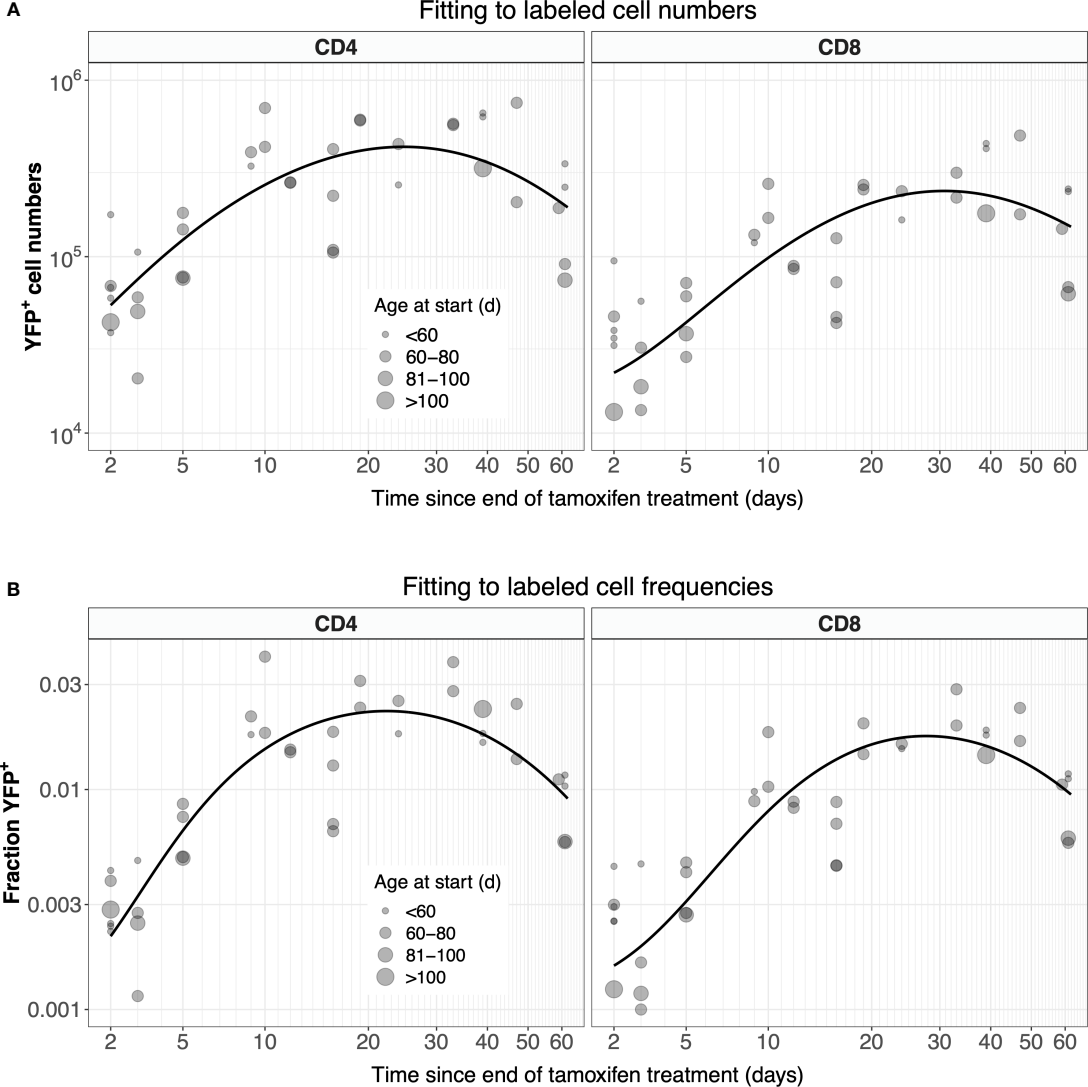
Modeling the dynamics of YFP^+^ naive CD4 and CD8 T cells after 5 days of tamoxifen treatment. **(A)** The timecourses of YFP^+^ cell numbers, recovered from spleen and lymph nodes, with the best-fitting model described by Equation 1. **(B)** Observed and fitted trajectories of YFP^+^ cell frequencies, modeled with Equation 2. Points are sized by the age class of the mouse at start of treatment.


Possibly due in part to the low efficiency of YFP induction, the measured numbers of labeled cells were somewhat noisy (**Figure 4A**, gray points). In studies involving flow cytometry of cells recovered from harvested tissues, estimates of subset frequencies typically exhibit smaller coefficients of variation than cell numbers. We therefore explored an alternative approach, modeling the kinetics of the proportion of naive cells that expressed YFP, *ℓ* = *L/N*, where *N* is the total number of naive CD4 or CD8 T cells, rather than of the kinetics of *L* (**Figure 4B**, gray points). Note however that these proportions also showed variation, again likely due to the low efficiency of YFP induction. We observed that total numbers of naive CD4 and CD8 T cells showed very little variation across the experiment (**Figures S2A, B**), and so we could reasonably assume that they were at or close to steady state (*dN/dt* = 0). In that case, Equation 1 can be recast as:

(2) $$\frac{d\ell }{dt}=\frac{\text{Θ}Y(t)}{N}-\mu \ell =\Theta Z\left(t\right)\;-\;\mu \ell ,$$

where *Z*(*t*) is the number of YFP + mSP cells normalized by the number of peripheral naive T cells, *N*.

The process of induction and loss of YFP^+^ cells in the thymus occurs along a chain of proliferating precursors leading back to lymphoid progenitor cells in the bone marrow. Rather than modeling this process explicitly, we described the timecourses of total *(Y(t))* and normalized *(Z(t))* YFP^+^ mSP cell numbers using the following empirical functions;


(3a) $$Y\left(t\right)=Y_{0}-at^{b}e^{-ct},$$

(3b) $$Z\left(t\right)=ke^{at}{\left(1+e^{v\left(t-\tau \right)}\right)}^{-\frac{a+b}{v}}.$$

For CD4 and CD8 T cells, Equation 3a and Equation 3b were fitted to their appropriate time-courses, using least squares and log- and logit-transformed YFP^+^ mSP numbers and frequencies, respectively (**Figure S4**). These fitted functions were then used in Equation 1 and Equation 2, which in turn were solved numerically and fitted to the observed time courses of numbers and frequencies of YFP^+^ naive T cells, respectively (**Figure 4**; see Methods for details). Our conclusions were insensitive to the precise functional forms of *Y(t)* and *Z(t)* – they were chosen simply to represent the expected and observed unimodal kinetic of labeled cells as they transit through the final stages of thymic development.

**Figure 5:**
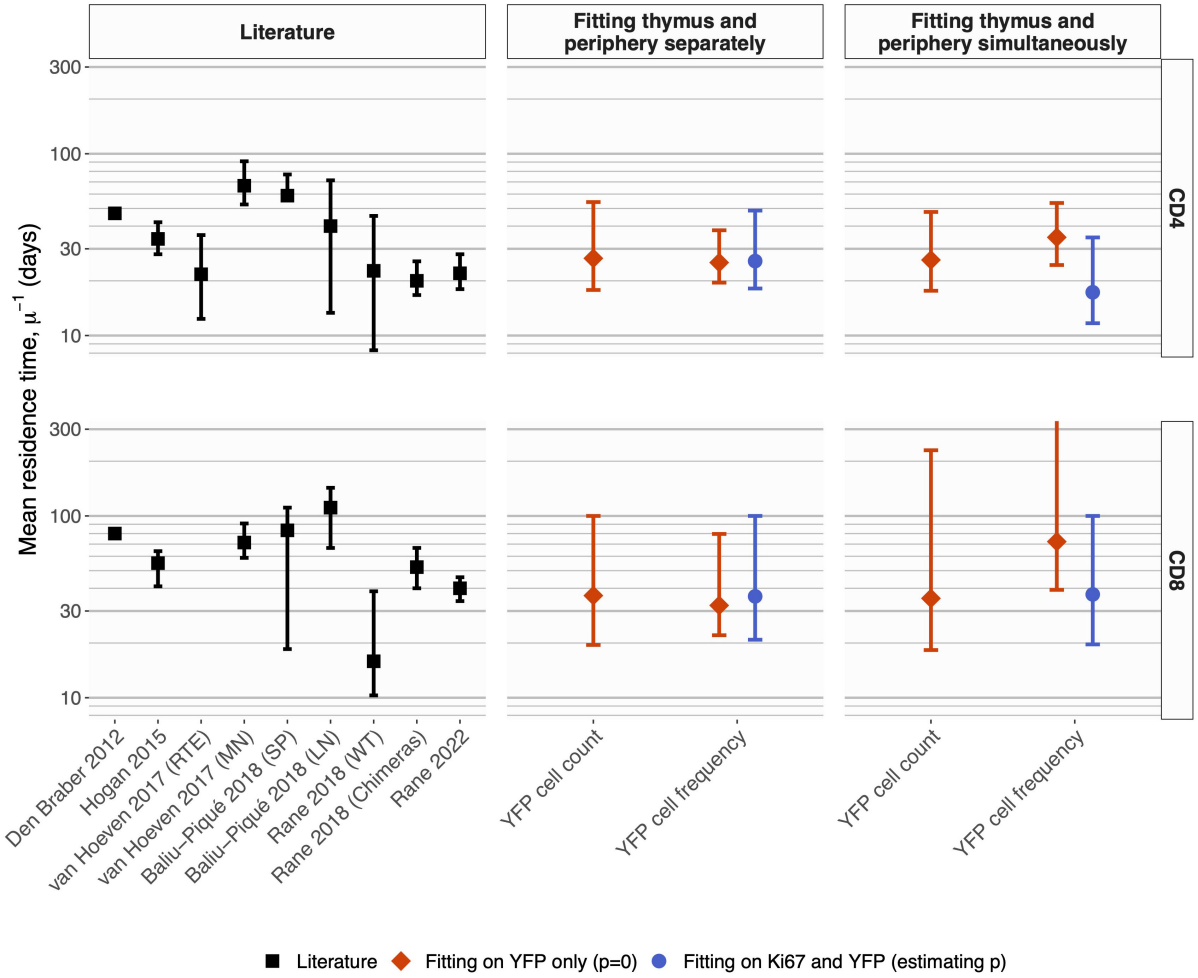
Summary of estimates of mean residence times of naive CD4 and CD8 T cells in adult mice, derived from different methods. Left panels; summary of published estimates. Van Hoeven et al. estimated the lifetimes of recent thymic emigrants (RTE) and mature naive (MN) T cells; they found these values to be indistinguishable for naive CD8 T cells. Values quoted by Rane (2018) (13) were derived from data from wild-type and thymectomized mice (‘WT’), and busulfan chimeric mice (‘Chimeras’). All estimates from Rane (2018) (13) and Rane (2022) (14) are for RTE and are lower bounds on the population-average residence time. Center panels; estimates derived from Ki67 reporter mice, using pre-determined, fitted empirical descriptions of the dynamics of labeled thymocytes as model inputs. Right panels; estimates derived from simultaneous fitting of the kinetics of labeled thymocytes and labeled naive T cells. In the center and right panels, red diamonds show estimates derived from the simpler model that assumes no proliferation among naive T cells; blue circular points show estimates derived from the larger model that explicitly tracks Ki67 within thymocytes and naive T cells and allows for naive T cell proliferation. Bars indicate 95% confidence intervals. One upper limit is truncated for clarity; see **Table 1** for all parameter estimates and confidence intervals.

The two methods yielded similar estimates of the mean residence time (1/*µ*) with comparable levels of uncertainty (**Figure 5**, center panels, diamond-shaped points). These estimates were consistent with those derived previously from BrdU or deuterium labeling (11, 27–29) and from busulfan chimeric mice (7, 13, 14), shown for reference in the left-most panel in **Figure 5**. Intuitively, residence times correspond roughly to the timescales of decline of the YFP labeling curves. These are upper bounds, because labeled cell numbers are sustained to some extent by residual influx from the thymus after tamoxifen treatment ends. The cell count and cell frequency methods also generated very similar estimates of the per capita rate of output of cells from the thymus (Θ; **Table 1**).

**Table 1:** Parameter estimates for all model fitting strategies.

Parameter	Model	Fit thymus & periphery	Method	CD4	CD8
Mean residence time (1/*μ*, days)	YFP only (*p* = 0)	Separately	Counts	18 (27-54)	36 (19-100)
	YFP only (*p* = 0)	Separately	Frequency	25 (20-38)	32 (22-80)
	YFP only (*p* = 0)	Simultaneously	Counts	26 (18-48)	35 (18-230)
	YFP only (*p* = 0)	Simultaneously	Frequency	35 (24-54)	72 (39-760)
	YFP + Ki67	Separately	Frequency	26 (17-50)	42 (22-100)
	YFP + Ki67	Simultaneously	Frequency	21 (15-43)	39 (20-100)
*Per capita* export rate (Θ, /day)	YFP only (*p* = 0)	Separately	Counts	0.73 (0.51-0.99)	0.92 (0.55-1.5)
	YFP only (*p* = 0)	Separately	Frequency	0.77 (0.55-0.99)	0.93 (0.58-1.3)
	YFP only (*p* = 0)	Simultaneously	Counts	0.74 (0.53-0.94)	1.00 (0.59-1.4)
	YFP only (*p* = 0)	Simultaneously	Frequency	0.59 (0.51-0.72)	0.63 (0.51-0.76)
	YFP + Ki67	Separately	Frequency	0.72 (0.50-0.99)	0.91 (0.54-1.4)
	YFP + Ki67	Simultaneously	Frequency	0.84 (0.54-1.0)	0.99 (0.59-1.4)
Mean interdivision time (1/*p*, days)	YFP + Ki67	Separately	Frequency	400 (240-930)	**2500 (520-10^4^**)^†^
	YFP + Ki67	Simultaneously	Frequency	**300 (200-850)** ^†^	**4600 (510-10^5^**)^†^
Duration of Ki67 expression (1/*β*, days)	YFP + Ki67	Separately	Frequency	2.0 (1.5-2.7)	4.3 (2.7-6.3)
	YFP + Ki67	Simultaneously	Frequency	1.7 (1.2-2.7)	4.3 (2.5-6.8)

Here we show parameters, with 95% confidence intervals, relating to naive CD4 and CD8 T cell dynamics in Ki67-reporter mice. These are derived from the simple model assuming no naive T cell division (‘YFP only (*p* = 0)’), or the full model that utilizes the live readouts of Ki67 expression to estimate *p* (‘YFP + Ki67’); from methods that fit thymic and peripheral labeling kinetics either separately or simultaneously; and from methods that model either the counts of YFP^+^ cells, or their frequencies within naive T cell populations. Estimates in bold and marked with †; in these models, adding *p* as a free parameter did not substantially improve the fit of the model over the nested model with *p* = 0 (see text); we quote them here for completeness.

We conclude that a simple homogeneous model without cell proliferation, and with pre-determined empirical functions describing the influx of labeled cells from the thymus, yields residence times of naive T cells consistent with those derived from other labeling methods. We also conclude that fitting on either the total numbers of YFP^+^ naive T cells, or their frequency, yields similar point estimates, and with comparable precision.

### Defining rates of naive T cell proliferation by combining YFP labeling with live readouts of Ki67 expression

The models above assume that YFP expression among naive T cells derives entirely from cells that divided in the thymus during tamoxifen treatment and were subsequently exported into the periphery. To generalize these models and allow for the possibility of naive T cell self-renewal, we returned to the full version of the model which describes the dynamics of Ki67 expression in mSP thymocytes and naive T cells in the periphery (**Figure 3**). Doing this would allow us to measure the contributions of intrathymic proliferation and self-renewal to the production of YFP^+^ naive T cells, and so to test the consensus view that naive T cells in mice divide rarely.

**Figure 6:**
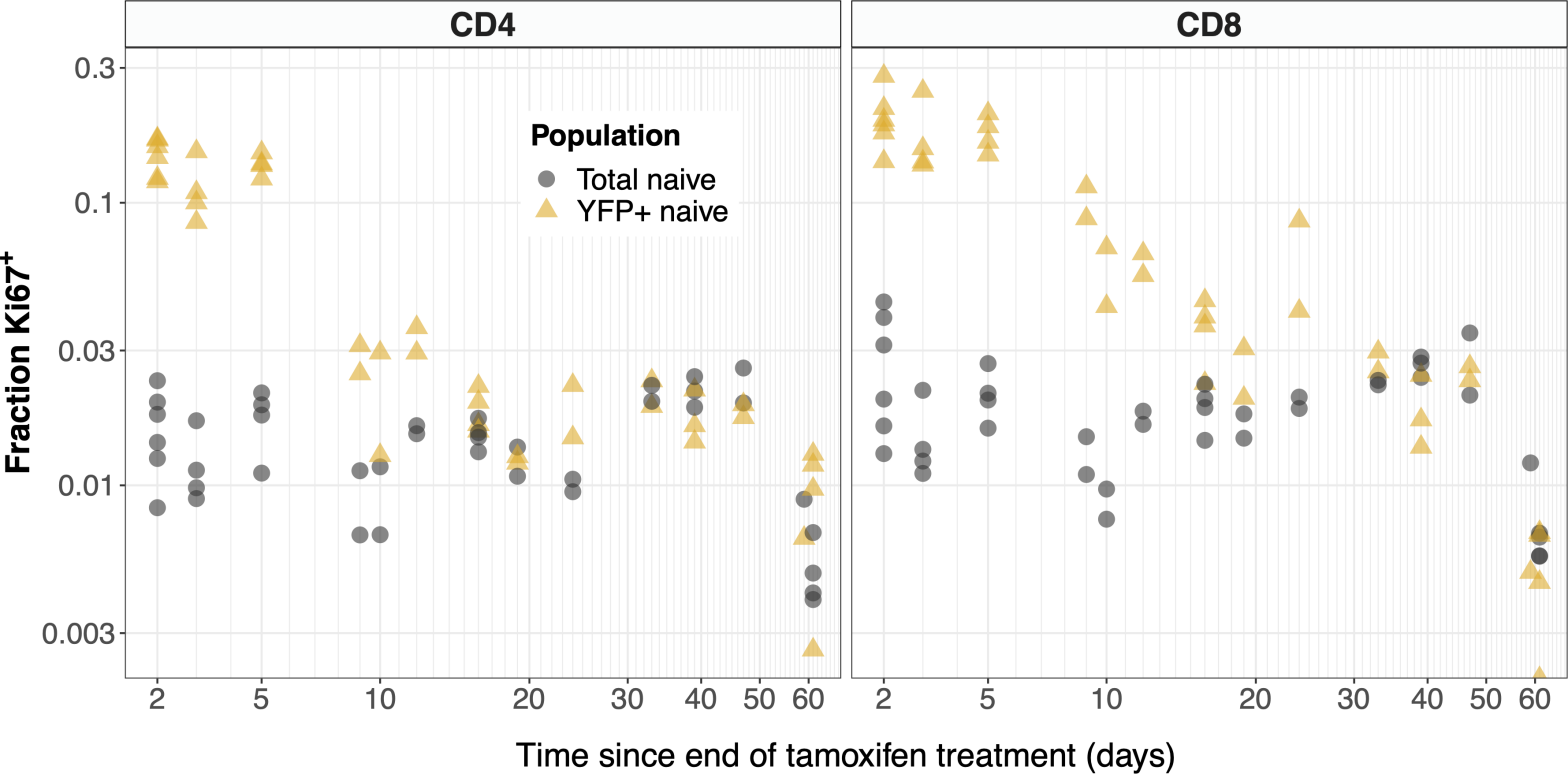
YFP labeled naive T cells are transiently enriched for recently-divided (Ki67^+^) cells. The observed frequencies of Ki67 expression among YFP^+^ (yellow) and total (gray) naive CD4 and CD8 T cells, after tamoxifen treatment.

The kinetics of Ki67 expression within YFP^+^ and total naive CD4 and CD8 T cells are shown in **Figure 6**. At early timepoints, we see that YFP-expressing naive T cells (yellow points) are significantly enriched for Ki67 relative to the population as a whole (gray). The observation is consistent with the intuitive result that YFP-expressing naive cells are predominantly recent thymic emigrants (RTE), many of which divided recently in the thymus, where levels of proliferation are high (**Figure S2I**). At later time points we see that Ki67 expression among naive YFP^+^ cells approaches the population average. This is perhaps counterintuitive; if naive T cells divide rarely, at late times we would expect all naive YFP^+^ cells to have lost any Ki67 deriving from intrathymic division. In contrast, we expect there to be substantial numbers of unlabeled RTE, with high levels of inherited Ki67. Therefore, long after treatment stops, we would expect the level of Ki67 expression among YFP^+^ cells to be lower than the population average. The convergence in frequencies has two potential explanations. One is that mature naive T cells do indeed divide at significant levels. The other is that YFP^+^ cells continue to be exported from the thymus long after tamoxifen treatment, perhaps due to labeling of stem-like precursor cells in the bone marrow. Indeed we did observe low but significant levels of label within the mSP4 and mSP8 populations more than 2 months after tamoxifen administration ended (**Figure S4**, points), which were captured by the functions we used to define these kinetics (**Figure S4**, black lines).

To estimate the rates of division of naive T cells, we expanded the basic model (Equation 1) to track Ki67^+^ and Ki67^−^ cells within the YFP^+^ population (*L* ^−^ and *L* ^+^, respectively; Equation 4). The model is shown schematically in **Figure 3**. Division induces Ki67 expression, and the return to Ki67^−^ is assumed to occur with first order kinetics at rate *β*. Thus 1/*β* is the mean residence time in the Ki67^+^ state, a compound parameter which is determined by both the intrinsic decay rate of the protein and the threshold of expression defining the boundary between Ki67^−^ and Ki67^+^ in the flow cytometric analysis. As before, we assume that YFP-labeling has no effect on cell dynamics, with all cells experiencing the same rates of division (*p*) and loss (*µ*);


$$\frac{dL^{+}}{dt}=\text{Θ}K^{+}\left(t\right)-L^{+}\left(\mu +\beta \right)+p\left(2L^{-}+L^{+}\right),$$

(4) $$\frac{dL^{-}}{dt}=\text{Θ}K^{-}\left(t\right)-L^{-}\left(\mu +p\right)+\beta L^{+}.$$

Here the empirical functions $K^{+/-}\left(t\right)$ describe the timecourses of the numbers of YFP^+^ Ki67 ^+/−^ mSP cells, respectively. We assume that Ki67^-^ and Ki67^+^ mSP cells are exported from the thymus at the same *per capita* rate Θ. Support for this assumption comes from the observation that in Rag-GFP reporter mice, Ki67 levels are similar among late stage SP thymocytes and GFP-bright recent thymic emigrants (14).

The measured values of *L^+^* and *L^−^* are not statistically independent; they derive from a single measurement of naive cell numbers using a cell counter, which is multiplied by the fractions of cells that are YFP^+^ Ki67^+^ and YFP^+^ Ki67^+^ respectively, estimated using flow cytometry. Any error or inter-mouse variation in the cell number will then be reflected in both *L^+^* and *L^−^*. To circumvent this, we recast Equation 4 to model the dynamics of two independent quantities; the total number of YFP^+^ cells, *L(t)*, and the proportion of these cells expressing Ki67, *ℓ* ^+^ *(t)* = *L^+^(t)/L(t)*;


$$\frac{dL}{dt}=\text{Θ}Y\left(t\right)+L\left(t\right)\left(p-\mu \right),$$

(5) $$\frac{d\ell^{+}}{dt}=\frac{\Theta }{L\left(t\right)}\left(K^{+}\left(t\right)-Y\left(t\right)\ell^{+}\left(t\right)\right)-\beta \ell^{+}+2p\left(1-\ell^{+}\left(t\right)\right).$$

The observed trajectories of the numbers of Ki67^+^ YFP^+^ mature SP thymocytes (*K^+^* (*t*)) were both well described by a unimodal function (**Figure S5**) which took the same form as the one used to describe the trajectories of YFP^+^ mSP4 or mSP8 numbers (*Y*(*t*), Equation 3a), but with distinct parameters. Using the fitted *K^+^* (*t*) and *Y(t)* as inputs, each with distinct parameters for CD4 and CD8 lineage thymocytes, we then fitted the model defined by Equation 5 to the observed time courses *ℓ^+^* (*t*) and *L*(*t*).

**Figure 7:**
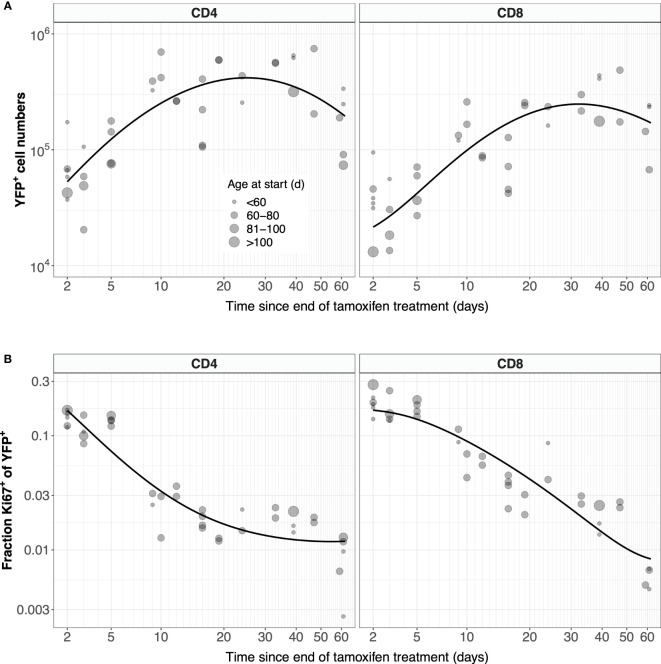
Models of naive T cell dynamics that explicitly track Ki67 expression. Shown are the fitted trajectories of YFP^+^ naive T cells **(A)** and the proportion of YFP^+^ cells that are Ki67^+^ **(B)**, modeled with Equation 5. Points are sized by the age class of the mouse at start of treatment.

Previous estimates of the naive T cell division rate *p* were very low, with mean interdivision times ranging from many months to years. Our point estimates were correspondingly small (400 days for CD4, 2500 days for CD8). Perhaps due to the relatively short chase period of 62 days, the mean interdivision times showed considerable uncertainty (**Table 1**). We therefore compared the quality of fits of the full model (Equation 5) to fits of the reduced and nested model with *p* = 0. Including *p* provided a substantial improvement for naive CD4 T cells (F-test; p-value 4.6 × 10^-4^), but not for CD8 (F-test; p-value 0.50). Fitted models with *p* free are shown in **Figure 7**, and for naive CD8 T cells were visually indistinguishable from the fits with *p* = 0. All parameters are shown in **Table 1**, with indications of the significance of adding *p*. Taken together, these analyses support the consensus that naive T cells in mice divide rarely, if at all (7, 10–13).

Again, the estimated residence times (**Figure 5**; central panels, blue circles) agreed with the estimates from the simpler model with no division (central panels, red diamonds), and with previous studies (**Figure 5**; left panels).

### Allowing for uncertainty in the label content of naive T cell precursors

**Figure 8:**
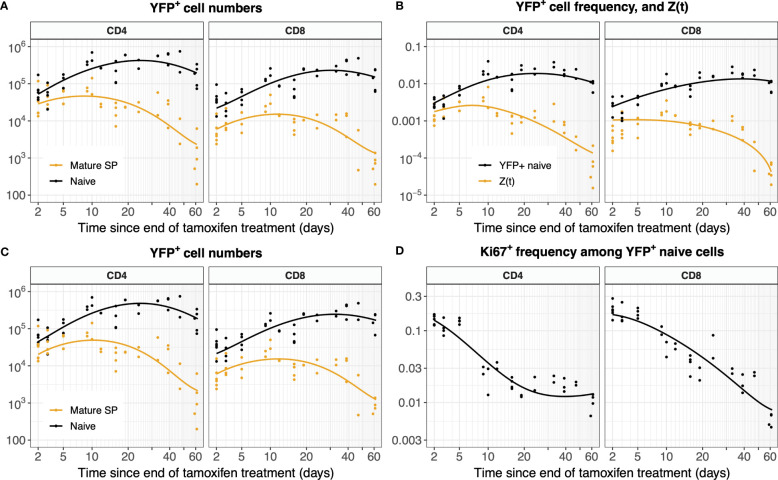
Models of YFP^+^ cell dynamics, fitted simultaneously to data from the thymus and the periphery. **(A)** Trajectories of YFP^+^ naive T cell numbers modeled with Equation 1 and of YFP^+^ mSPs modeled with Equation 3a. **(B)** Trajectories of the YFP^+^ frequency within naive T cells modeled with Equation 2, and of the ratio of YFP^+^ mSP to naive T cell numbers (*Z*(*t*), Equation 3b). **(C)** Numbers of YFP^+^ naive T cells and YFP^+^ mature SP thymocytes (*L*(*t*) and *Y*(*t*), Equation 5). **(D)** Fraction of YFP^+^ naive cells expressing Ki67 (*ℓ* ^+^(*t*), Equation 5).

The analyses above assumed that the time courses of YFP^+^ mSP cells in the thymus were described precisely by the fitted functions *Y*(*t*) and *Z*(*t*). Intuitively, neglecting uncertainty in these functions may lead us to overestimate our confidence in the parameters describing naive T cell dynamics. To explore this issue, we fitted the labeling data from the thymus and periphery simultaneously. We did this for the simple model assuming no division, fitting on both YFP counts (**Figure 8A**) and frequencies (**Figure 8B**). We also performed simultaneous fitting for the full model incorporating Ki67 expression (Equation 5), which yielded the predicted numbers of YFP^+^ naive T cells (**Figure 8C**) and the proportion expressing Ki67 (**Figure 8D**).

Allowing for uncertainty in the parameters defining the thymic source functions by fitting them simultaneously with the peripheral dynamics increased the uncertainty in residence times derived from the simplest model, but had relatively little effect on residence times for the models constrained by the additional information from Ki67 (**Figure 5**, **Table 1**). However, the mean interdivision time became substantially more uncertain for CD8 T cells when fitting thymus and periphery simultaneously (**Table 1**). Indeed, when we compared the quality of fits of each model to its nested counterpart with no peripheral self-renewal (*p*=0), the models with *p*=0 had strongest support, for both CD4 and CD8 T cells (F-test; p-values of 0.17 and 0.88, respectively).

### Dynamics of YFP-labeled cells are representative of the whole population

To further validate the system, we tested the assumption that YFP-expressing cells behave identically to the population as a whole. To do this we used the parameters derived from the best fitting models of YFP-expressing cell dynamics to predict Ki67 expression among bulk naive T cells, given its expression among their mSP precursors in the thymus.

We took advantage of the pairing of measurements of thymocytes and peripheral naive T cells in each mouse. We expect the total number of Ki67^+^ naive CD4 or CD8 T cells in spleen and lymph nodes at a time t , $N_{\text{pred}}^{+}\left(t\right)$ , to be approximately the number of Ki67^+^ cells exported from the thymus in the previous 1/*β* days, plus two times the number of naive T cells that divided during the same time period;


(6) $$N_{pred}^{+}=\frac{\Theta K_{\text{mSP}}^{+}}{\beta }+\frac{2\mathrm{pN}}{\beta }$$

This can be expressed as the predicted Ki67^+^ fraction within the total naive pool. As described above, this fraction is the average of that observed in spleen and lymph nodes, weighted by the numbers of cells in each;


(7) $$k_{pred}^{+}=\frac{N_{pred}^{+}}{N}=\frac{\Theta K_{\text{mSP}}^{+}}{\beta N}+\frac{2p}{\beta }$$

This approximation assumes (i) *p* is small compared to the rate of loss of Ki67 (*β*), (ii) the duration of Ki67 expression is much shorter than the naive T cell lifespan, so that we do not need to account for any loss of divided cells during this period, and (iii) that Ki67^+^ mSP cell numbers are constant over the previous 1/*β* days.

We then generated a point estimate of $k_{\text{pred}}^{+}$ for each mouse, using its observed values of $K_{\text{mSP}}^{+}$ and *N*, and the population-average estimates of the parameters generated by simultaneously fitting the kinetics of thymic and peripheral YFP-labeled cells. We generated 95% confidence intervals on each estimate of $k_{\text{pred}}^{+}$ by sampling from the bootstrap estimates of these parameters, and compared these predictions to the observed Ki67^+^ fraction for each mouse,


(8) $$k_{\text{obs}}^{+}=\frac{N^{+}}{N}.$$

**Figure 9:**
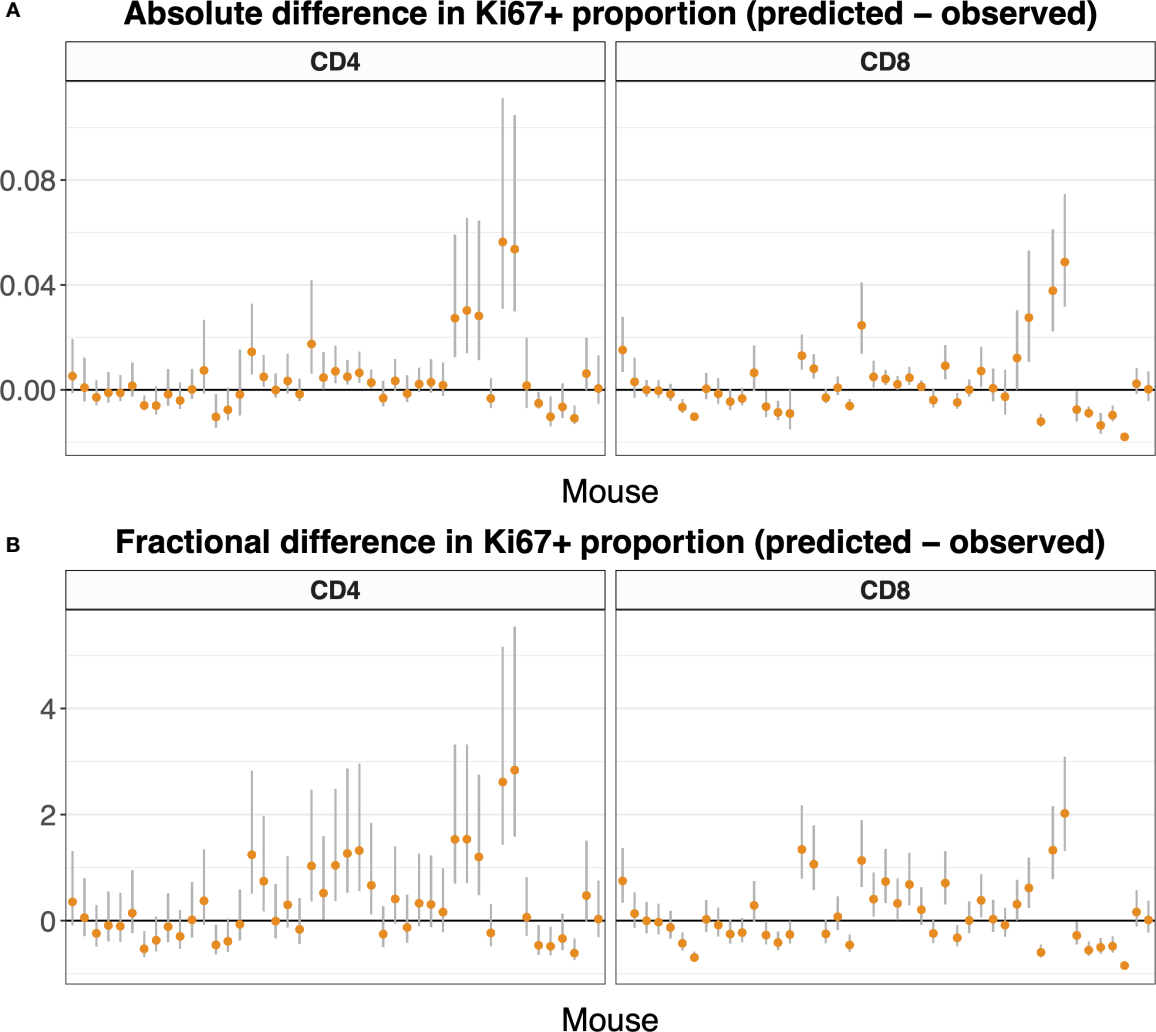
Difference between predicted and observed Ki67 expression on naive T cells, by mouse, calculated using Equation 7, and presented in both absolute **(A)** and fractional **(B)** terms. We used the parameters and 95% confidence intervals derived from the fitted model Equation 5 along with Equation 3a and *K* ^+^(*t*). For both naive CD4 and CD8 T cells, allowing the self-renewal rate *p* to be non-zero did not improve the fit substantially over a reduced model with *p* = 0 (see text). Here we present the Ki67 expression predictions with *p* = 0; predictions including the small effect of non-zero *p* are very similar (**Figure S6**). Mice are ordered by time post-treatment.

**Figure 9** shows these comparisons, with mice ordered by time since the end of tamoxifen treatment. It was generated using the statistically favored version of the model in Equation 5, in which the naive T cells do not divide (*p* = 0). **Figure S6** shows the comparisons using the full model in which *p* was estimated, which are similar.

In both cases, the differences between predicted and observed frequencies were small in both absolute and relative terms (**Figures 9A, B**, respectively; see also **Figure S6**), although there was a trend for the models to slightly overestimate Ki67 expression in the periphery. Discrepancies could potentially be due to uncertainty in gating Ki67 expression in flow cytometry, and/or small differences in expected lifespans of very recent thymic emigrants and more mature naive cells, differences that our experiment was not designed to resolve. Since division rates are so low, our analyses confirmed that the bulk of Ki67 expression in the periphery derives from newly generated naive T cells that divided shortly before leaving the thymus. Our estimates of the Ki67 lifetime are somewhat lower than those established previously (**Table 1**), consistent with a time lag before Ki67-expressing naive T cells appear in the periphery. In summary, the predictive ability of the model combined with our finding that the estimated lifespans of naive CD4 and CD8 T cells derived from this system are compatible with previous estimates, and the observation that cell numbers and Ki67 levels are indistinguishable in tamoxifen-treated reporter mice and WT controls (**Figure S3**), support the conclusion that the induction of YFP has no appreciable effect on cellular dynamics.

## Discussion

We have shown here that Ki67 reporter mice can be used to study cell division and turnover, in a manner conceptually similar to BrdU and deuterium labeling. Each has its limitations; BrdU is toxic when administered for extended periods, and one must model its dilution by subsequent divisions in the chase period. Deuterium labeling has the key advantage of being safe to perform in humans but, because the readout is the enrichment for deuterium in DNA strands pooled from a sample of the population of interest, one must calibrate to a fast-dividing population with high enrichment to infer the proportion of DNA strands that contain deuterium. Further, most labeling studies assume that the source population is dividing rapidly and so 100% labeled during the pulse phase, and switches to unlabeled as soon as the chase period begins. It is unclear that this assumption always holds, but for many cell populations the true precursor may be uncertain, and so verifying its label content may not be possible. A feature of our Ki67 reporter system is the low efficiency of induction per cell division, which makes it necessary to both know the precursor population and explicitly (here, empirically) model the kinetics of its label content. We also saw that YFP expression persisted at a low level among thymocytes well after tamoxifen administration had ended, likely due to heritable labeling within self-renewing lymphocyte progenitors. The resulting residual influx of YFP-expressing naive T cells needed to be accounted for when modeling the chase period.

Ki67 expression levels have been used in previous studies modeling cellular dynamics, but largely for validation purposes as a correlate of proliferative activity measured by other means (28, 30). In this study, as in our previous work (13, 14, 17, 31) we model Ki67 expression explicitly; here, to inform estimates of rates of thymic export and of naive T cell self-renewal. We have shown previously that most Ki67-expressing naive T cells in mice emigrated from the thymus within the previous few days, and we and others have shown that there is very little self-renewal of more mature naive T cells. Our analyses are consistent with this conclusion.

The fitting methods we explored here yielded consistent estimates of the *per capita* rates of release of mature SP4 and SP8 cells from the thymus, Θ, of approximately 0.7 and 1 day^−1^ respectively. The adult mice in this experiment had approximately 10^6^ and 3 x 10^5^ mSP4 and mSP8 thymocytes, implying that roughly 7 x 10^5^ naive CD4 and 3 x 10^5^ naive CD8 T cells are exported from the thymus each day. These point estimates align closely with our previous estimates of 5 x 10^5^ and 2.4 x 10^5^ respectively (7), and with an older estimate of a total daily export rate of 10^6^ naive T cells (26).

The low efficiency of induction of YFP per cell division in this mouse model allows it in principle to be used as a division counter; the YFP expression within a closed population reflects the average number of divisions it went through during tamoxifen administration (**Figure 2C**). Our system has some similarities to the one described by Bresser et al. (32), in which a fluorescent reporter construct is switched on with a low probability per division. To follow specific populations the reporter construct is induced with a retrovirus and cells are adoptively transferred into a congenic host, where the accumulation of reporter expression can be tracked over time. The system we study here does not require adoptive transfer and so can be used to examine unperturbed cellular dynamics at steady state, as well as endogeneous responses to immune stimuli.

Our study has several limitations. When we stratified labeled cell dynamics by Ki67 expression and fitted thymic and peripheral dynamics simultaneously, we found the strongest support for models in which naive T cells do not divide. Simpler analyses in which the influx of labeled cells from the thymus was described with a fixed and predetermined function supported self-renewal within naive CD4, but not CD8, T cells. Our study design made it difficult to resolve division rates unequivocally, because the duration of the chase period (62 days) was substantially shorter than the interdivision times estimated by us and others. Another limitation is that the study was not designed to model the known effects of post-thymic cell age on turnover (13, 14, 33). However, within the range of mouse ages we studied, and with the short chase period, we expect this cell age effect to be small; and in any case, our estimates of residence times span those from reports that isolated the residence times of naive T cells of different ages (**Figure 5**). Perhaps the major limitation is that due to the low efficiency of YFP induction in this reporter strain, the numbers and frequencies of YFP expressing naive T cells were small. Noise in these observations contributed to the uncertainty in residence times. Consequently, this reporter may be better suited for studying populations that divide extensively, such as T cells responding to antigen.

In summary, the Ki67 reporter system allows the long-term tracking of the fate of cells that divided within a given time window, and can be used as a means of quantifying cell population dynamics in a manner analogous to (and consistent with) DNA labeling assays. The low efficiency of induction in this particular construct requires one to explicitly model the dynamics of reporter expression in the precursor of the population of interest. This requirement may be turned to an advantage in settings in which differentiation pathways are uncertain. By fitting the labeling data using different candidate precursors, each with empirically described time courses of label content, one could then use model selection tools to reveal the relative support for different pathways.

## Methods

### Experimental mouse model

*Ki67-mCherry-CreER* mice have been previously described (5). Briefly, C57Bl6/J mice underwent targeted replacement of the terminal exon 14 of the *Mki67* locus with a modified exon 14 sequence with upstream FRT flanked neomycin cassette, and downstream mCherry fusion construct, IRES sequence and CreERT2 cDNA. Mice were crossed with *actin-FLPe* mice to excise the neomycin selection cassette, before crossing with *Rosa26^RYFP^* strain (34), to generate *Ki67-mCherry-CreER Rosa26^RYFP^* double reporter mice. Cre recombinase activity was induced *in vivo* in these mice following i.p. injection with 2mg of tamxoxifen (Sigma) diluted in corn oil (Fisher Scientific) for five consecutive days. *Ki67-mCherry-CreER Rosa26^RYFP^* double reporter mice were bred at Charles River U.K. Ltd and the Comparative Biology Unit, Royal Free Hospital. Experiments were performed according to the UCL Animal Welfare and Ethical Review Body and Home Office regulations.

### Flow cytometry

Single cell suspensions were prepared from the thymus, spleen and lymph nodes (cervical, axillary, brachial, inguinal, and mesenteric) of *Ki67-mCherry-CreER Rosa26^RYFP^* double reporter mice. Cells were stained with the following monoclonal antibodies and cell dyes: TCR-β APC, CD4^+^ PerCP-eFluor710, CD44 APC-eFluor780, CD25 PE, CD25 eFluor450, CD25 PE-Cy7, CD62L eFluor450 (all eBioscience), TCR- β PerCP-Cy5.5 CD4^+^ BV711, CD44 BV785, CD25 BV650 (all Biolegend), CD62L BUV737 (BD Biosciences), LIVE/DEAD nearIR and LIVE/DEAD blue viability dyes. Cells were acquired on a BD LSR-Fortessa flow cytometer and analyzed with Flowjo software (Treestar). Gating strategy to identify DP, mSP and naive peripheral T cells was identical to that we have employed previously for analysis of mice expressing Ki67-RFP fusion protein (14). Briefly, DP thymocytes are identified as CD4^+^CD8^+^ CR^lo^ CD5^lo^, CD4 mSP are CD4^+^CD8^−^CD44^lo^CD25^lo^CD62L^hi^, CD8 mSP are CD4^−^CD8^+^CD44^lo^TCR^hi^CD62L^hi^, peripheral naive CD4^+^T cells are TCRR^hi^CD4^+^CD44^lo^CD62L^hi^, and peripheral naive CD8^+^T cells are TCR^hi^CD8^+^CD44^lo^CD62L^hi^.

### *In vitro* T cell stimulations

Total lymph node cells from *Ki67-mCherry-CreER Rosa26^RYFP^* mice were labeled with 2 *μ* M of CellTraceTM Violet (Invitrogen) in Dulbecco’s PBS (Invitrogen) for 10 min at 37°C and washed twice. Cells were cultured at 37°C with 5% CO2 in RPMI-1640 (Gibco, Invitrogen Corporation, CA) supplemented with 10% (v/v) fetal bovine serum (FBS) (Gibco Invitrogen), 0.1% (v/v) 2-mercaptoethanol *β* ME (Sigma Aldrich) and 1% (v/v) penicillin-streptomycin (Gibco Invitrogen) (RPMI-10). Cells were activated by CD3 and CD28 mAb, at concentrations indicated, bound to 96 well flat bottom plates overnight at 4°C and washed with PBS prior to culture. Cells were cultured at 10uperscript6/ml in 200 *μ*l. 4OH-tamoxifen (Sigma) was added to cultures at the indicated concentrations. Cells were recovered at the indicated times, and analyzed by flow cytometry for expression of YFP and mCherry, and stained for expression of TCR.

### Model fitting

All fitting was performed using the FME wrapper within Grind in *R* version 4.1.2, using the implemented pseudorandom-search algorithm on log- or logit-transformed observations. All data and code required to reproduce the analyses and figures can be obtained at https://github.com/andrewjyates/Lukas-Ki67YFP.git.

## Data availability statement

All the data used in this study, together with annotated code for performing the analyses and generating figures, are available at https://github.com/andrewjyates/Lukas-Ki67YFP.git.

## Ethics statement

The animal study was reviewed and approved by the UCL Animal Welfare and Ethical Review Body and the UK Home Office.

## Author contributions

EL: Analysis, drafting manuscript. TH and CW: Performed experiments. BS: Supervised experiments, conceptualized study. AY: Supervised and performed analyses, conceptualized study. All authors contributed to the article and approved the submitted version.

## Supplementary material

Supplementary Figure 1 Distribution of the ages of mice at initiation of tamoxifen treatment. Median age indicated with the blue line.

Supplementary Figure 2 First row: Measured numbers of naive T cells in lymph nodes **(A)**, spleen **(B)** and of mature single positive thymocytes **(C)**. Second row: Frequency of YFP expression in naive T cell populations in lymph nodes **(D)**, in spleen **(E)** and in mature single positive thymocytes **(F)**. Third row: Frequency of Ki67 expression in naive T cell populations in lymph nodes **(G)**, in spleen **(H)** and in mature single positive thymocytes **(I)**. **(J)** Ki67 expression among naive T cells in spleen and lymph nodes is strongly correlated.

Supplementary Figure 3 Naive T cell numbers and Ki67 expression frequencies in tamoxifen-treated Ki67 reporter mice and wild-type controls. Upper row shows total number of naive CD4 and naive CD8 T cells recovered from LN and spleen of either tamoxifen treatedKi67 reporter mice (TAM), control reporter mice not given tamoxifen (NO TAM) and WT control mice, at the indicated age of culling (S1K). Bottom row shows the frequency of Ki67 reporter detected in the corresponding naive LN T cell population of either tamoxifen treated Ki67 reporter mice (TAM) or control reporter mice not given tamoxifen (NO TAM).

Supplementary Figure 4 Phenomenological functions describing the timecourses of YFP-expression among mature SP cells in the thymus after tamoxifen treatment. **(A)** Best fits of the time-varying numbers of YFP^+^ mSPs (*Y*(*t*); Equation 3a). **(B)** The ratio of YFP^+^ mSPs to naive T cell numbers (*Z*(*t*); Equation 3b).

Supplementary Figure 5 Numbers of Ki67-expressing fluorescent mSPs over time, modeled emprically with *K* ^+^(*t*) = *K*_0_ + *at^b^e^-ct^*.

Supplementary Figure 6 Difference between predicted and observed Ki67 expression on naive T cells, by mouse, calculated using Equation 7, and presented as in both absolute **(A)** and fractional **(B)** terms, using parameters derived from models in which the proliferation rate p was allowed to be non-zero. Mice are ordered by time post-treatment.
