# Supplementary figures and images for: Quantifying cellular dynamics in mice using a novel fluorescent division reporter system
Source: Front Immunol. 2023 Jul 27;14:1157705. doi: 10.3389/fimmu.2023.1157705 (PMC10412932; doi:10.3389/fimmu.2023.1157705)

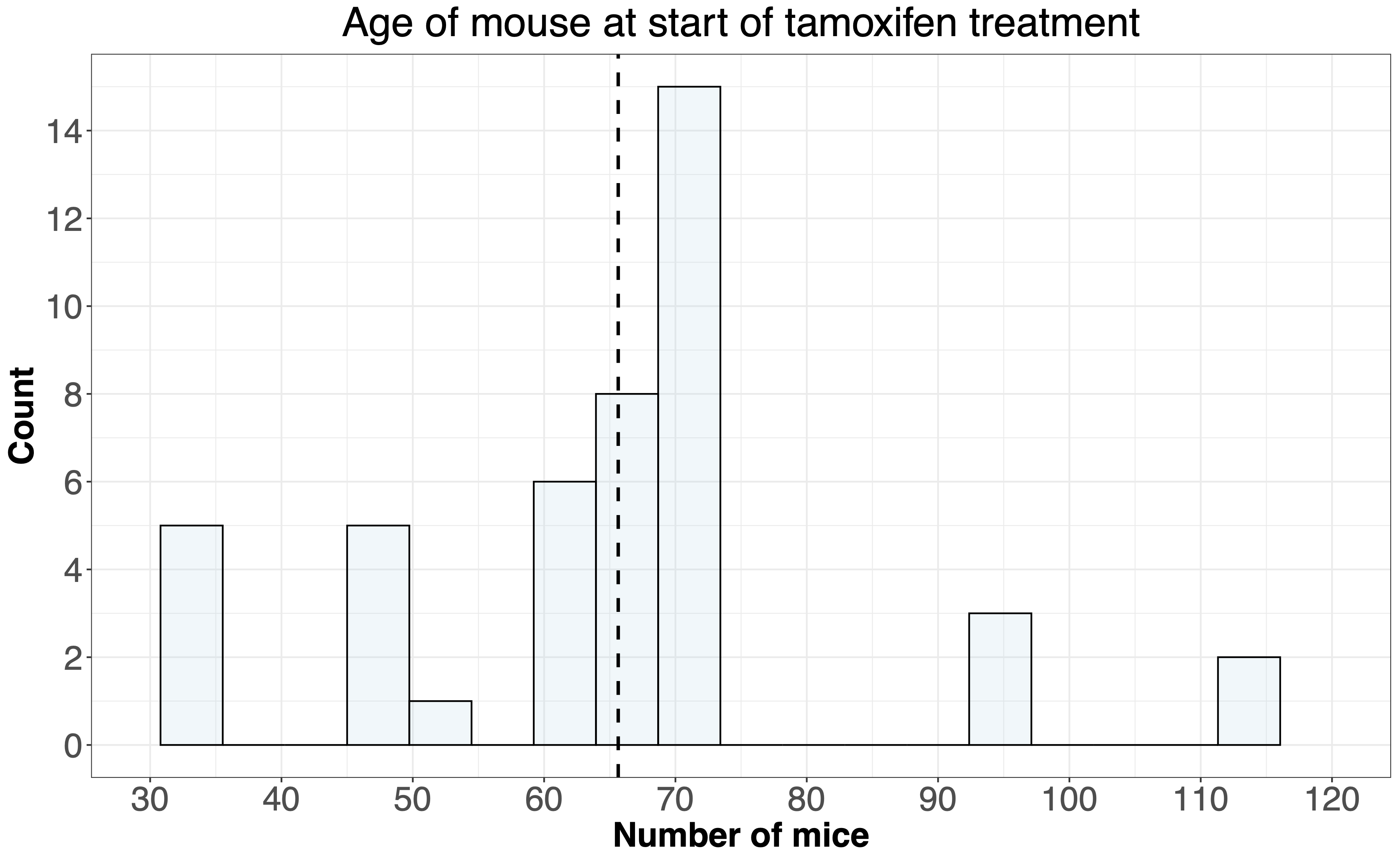

Supplement: Supplementary Figure 1 — Distribution of the ages of mice at initiation of tamoxifen treatment. Median age indicated with the blue line. [file DataSheet_1.zip › Figure-S1.tiff]

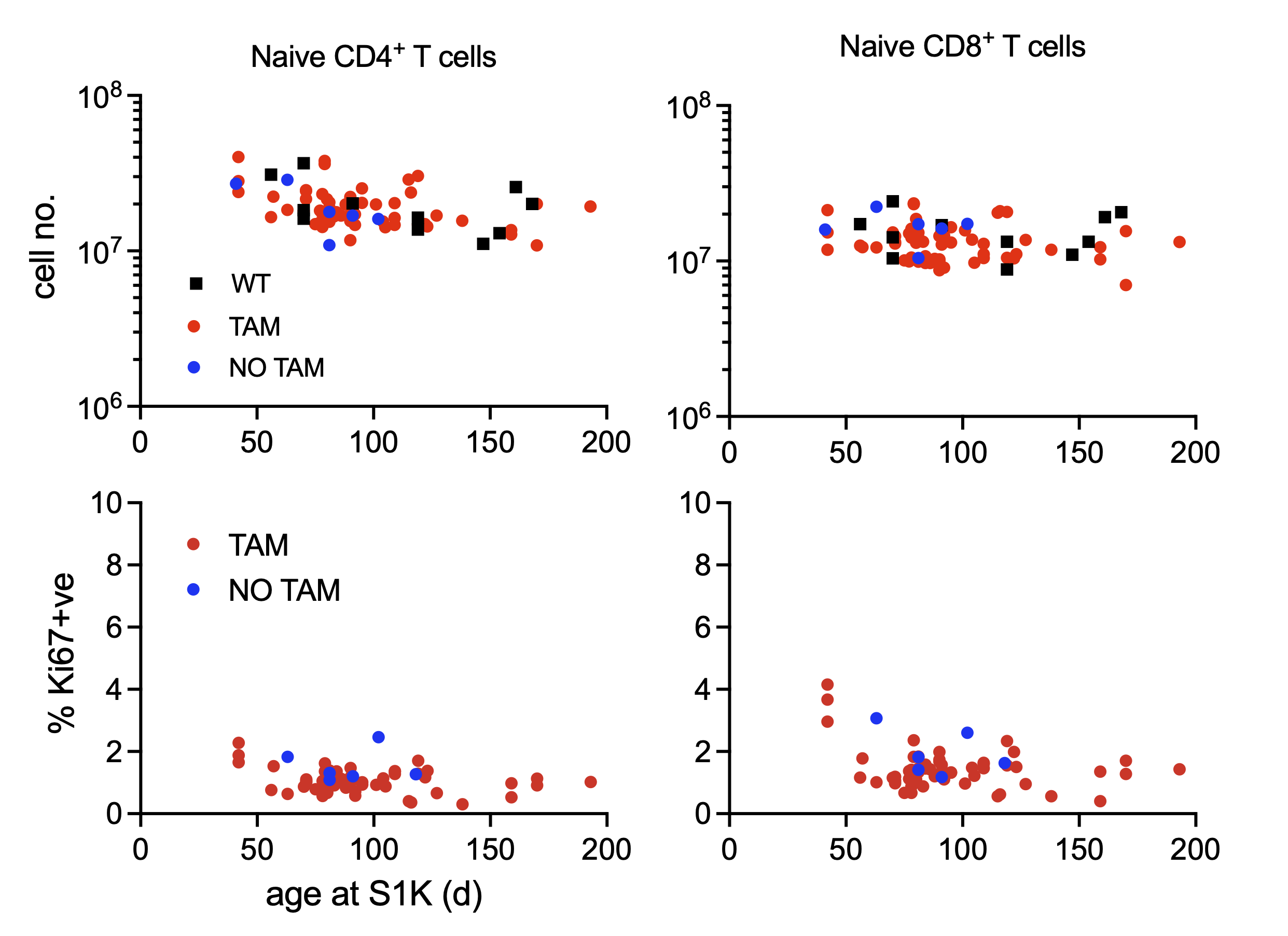

Supplement: Supplementary Figure 1 — Distribution of the ages of mice at initiation of tamoxifen treatment. Median age indicated with the blue line. [file DataSheet_1.zip › Figure-S3.tiff]

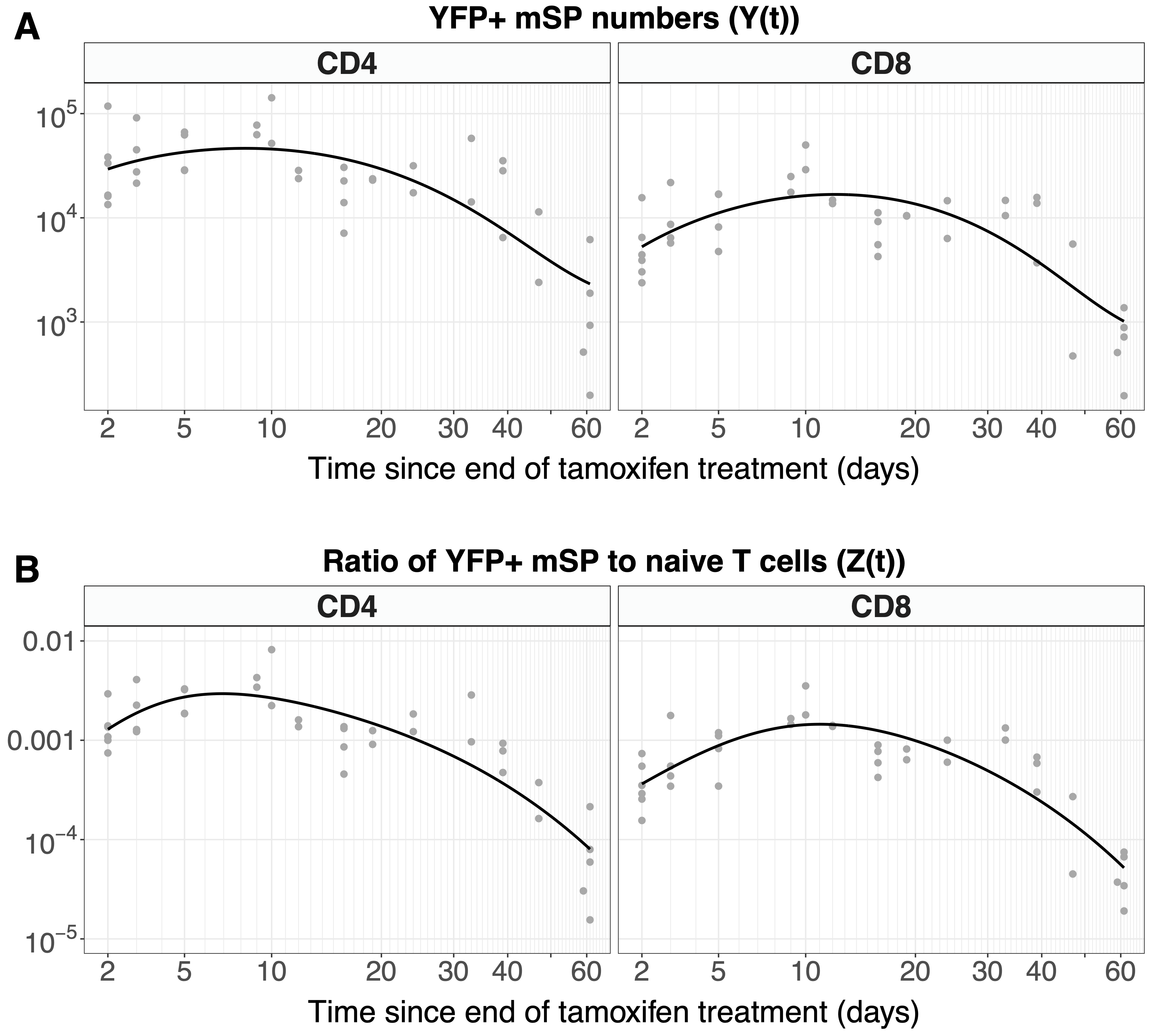

Supplement: Supplementary Figure 1 — Distribution of the ages of mice at initiation of tamoxifen treatment. Median age indicated with the blue line. [file DataSheet_1.zip › Figure-S4.tiff]

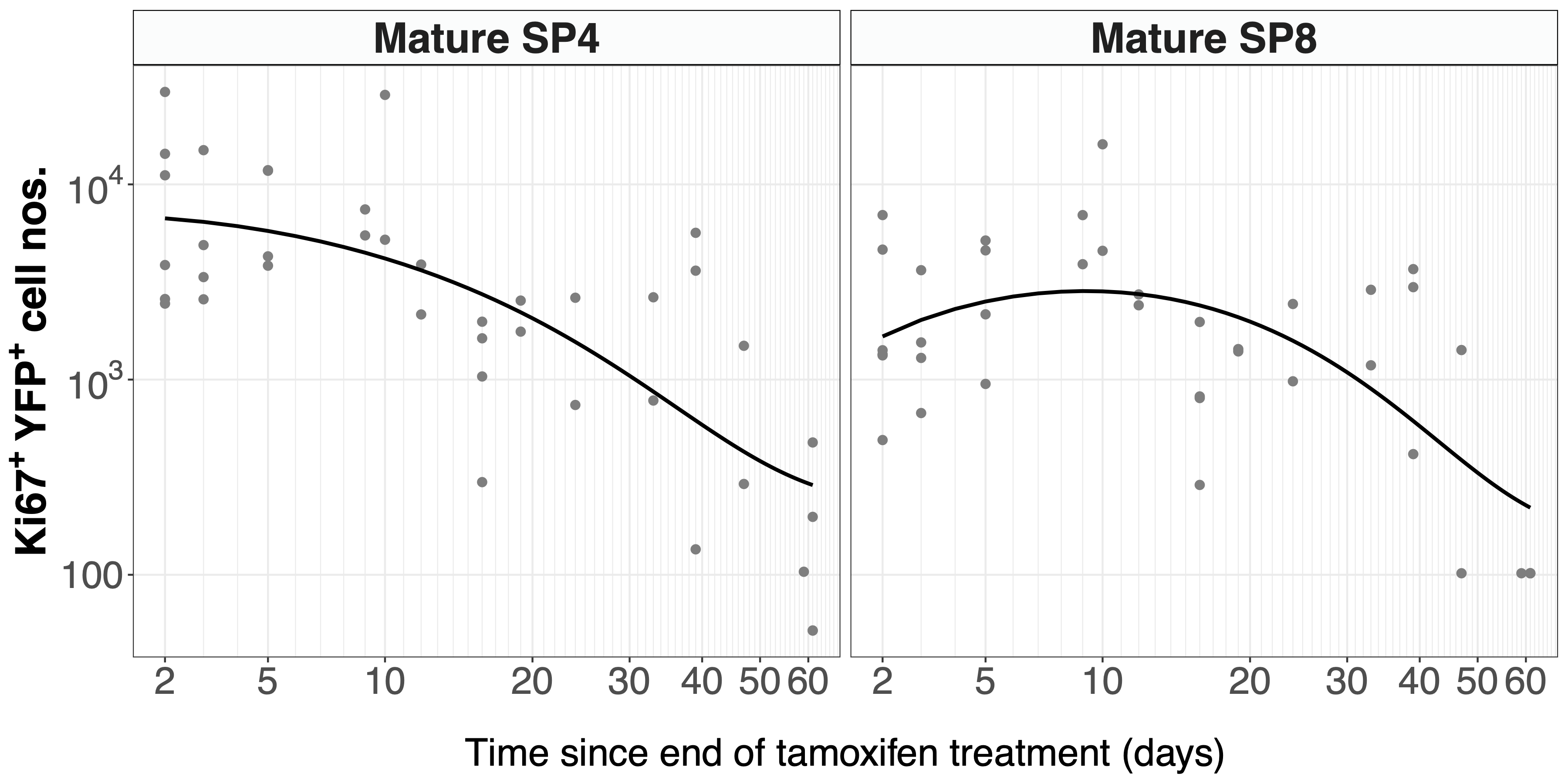

Supplement: Supplementary Figure 1 — Distribution of the ages of mice at initiation of tamoxifen treatment. Median age indicated with the blue line. [file DataSheet_1.zip › Figure-S5.tiff]

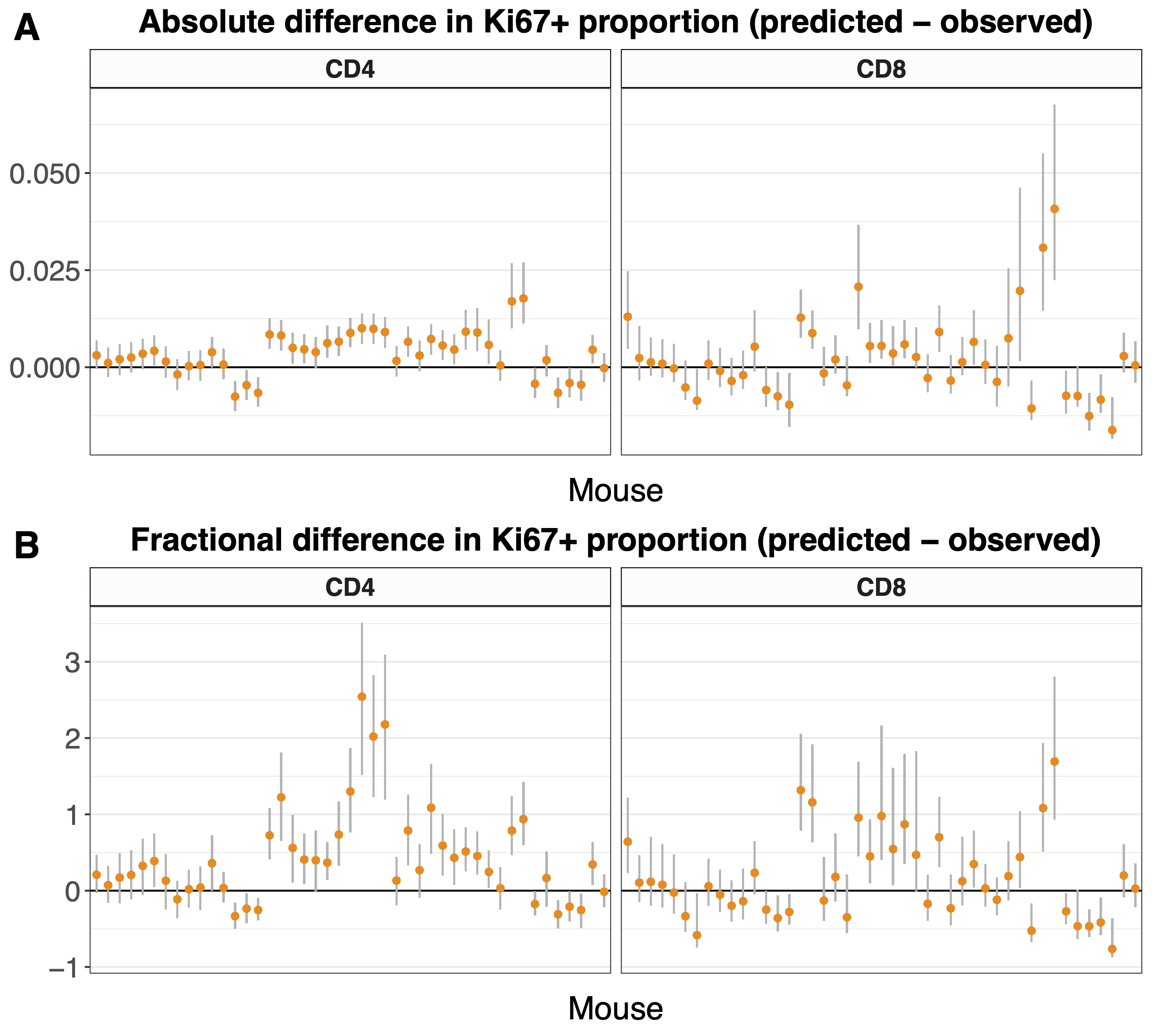

Supplement: Supplementary Figure 1 — Distribution of the ages of mice at initiation of tamoxifen treatment. Median age indicated with the blue line. [file DataSheet_1.zip › Figure-S6.tiff]
